# In Vitro Anti-Inflammatory Activity of Peptides Obtained by Tryptic Shaving of Surface Proteins of *Streptococcus thermophilus* LMD-9

**DOI:** 10.3390/foods11081157

**Published:** 2022-04-16

**Authors:** Rania Allouche, Zeeshan Hafeez, Florent Papier, Annie Dary-Mourot, Magali Genay, Laurent Miclo

**Affiliations:** CALBINOTOX, Université de Lorraine, F-54000 Nancy, France; zeeshan.hafeez@univ-lorraine.fr (Z.H.); florent.papier@univ-lorraine.fr (F.P.); annie.dary@univ-lorraine.fr (A.D.-M.); magali.genay@univ-lorraine.fr (M.G.); laurent.miclo@univ-lorraine.fr (L.M.)

**Keywords:** *Streptococcus thermophilus*, cell surface proteins, tryptic hydrolysis, anti-inflammatory

## Abstract

*Streptococcus thermophilus*, a lactic acid bacterium widely used in the dairy industry, is consumed regularly by a significant proportion of the population. Some strains show in vitro anti-inflammatory activity which is not fully understood. We hypothesized that peptides released from the surface proteins of this bacterium during digestion could be implied in this activity. Consequently, we prepared a peptide hydrolysate by shaving and hydrolysis of surface proteins using trypsin, and the origin of peptides was checked by liquid chromatography–tandem mass spectrometry (LC-MS/MS) analysis. Most of the identified peptides originated from bacterial cell surface proteins. The anti-inflammatory activity of peptide hydrolysate was investigated under inflammatory conditions in two cell models. Peptide hydrolysate significantly decreased secretion of pro-inflammatory cytokine IL-8 in lipopolysaccharide (LPS)-stimulated human colon epithelial HT-29 cells. It also reduced the production of pro-inflammatory cytokines IL-8, IL-1β and the protein expression levels of Pro-IL-1β and COX-2 in LPS-stimulated THP-1 macrophages. The results showed that peptides released from bacterial surface proteins by a pancreatic protease could therefore participate in an anti-inflammatory activity of *S. thermophilus* LMD-9 and could prevent low-grade inflammation.

## 1. Introduction

Inflammation is a part of the regular host reaction to injury or infection caused by pathogens, damaged cells, irritants or allergens. However, low-grade chronic inflammation is often associated with pathologies such as type 2 diabetes, inflammatory bowel disease (IBD), arthritis or atherosclerosis and other cardiovascular diseases [1,2]. Recent studies showed that inflammation also favors the development of severe forms of COVID-19 [3,4]. Although these diseases are generally treated by pharmaceutical approaches/drugs [5,6], a long-term use of such drugs can trigger side effects that can impair health. Numerous nutritional studies have highlighted that natural food sources containing bioactive peptides/molecules could modulate inflammation key factors and consequently delay the onset of these chronic diseases [6,7]. Bioactive peptides are defined as peptide sequences, derived from food proteins, which exert a beneficial effect on consumer health beyond the nutritional effect [8]. They can be released from these proteins by enzymatic proteolysis in vitro and/or in vivo or by lactic acid bacteria (LAB) or yeasts during the fermentation process [9] and, having thus become physiologically active, can regulate diverse physiological functions [10]. Moreover, LAB that can be a part of some food products, since they are widely used for the manufacture of fermented dairy products, have also been reported to display beneficial health effects such as anti-inflammatory properties both in in vitro and in vivo studies [11,12,13].

Among the LAB, *Streptococcus thermophilus*, belonging to the commensal, opportunistic and pathogenic species of the streptococcus genus, is the only species to be used in the food sector [14] as it is considered as GRAS (Generally Recognized As Safe) by the FDA and has obtained QPS (Qualified Presumption of Safety) status from EFSA. It is widely employed as a secondary starter culture in the dairy industry for milk acidification by transforming lactose into lactic acid [15,16], and thus contributes to both the fermentation and flavoring of dairy products. Besides its excellent technofunctional properties, *S. thermophilus* is capable of generating a variety of peptides with diverse bioactivities from milk proteins via its surface proteolytic system [17,18], whose main actor is cell envelope proteinase PrtS. Furthermore, *S. thermophilus* beneficial health effects have been reported in many studies [19]: alleviation of lactose intolerance, prevention of gastritis as well as of infectious diarrhea. It has several probiotic characteristics, especially producing biologically active molecules, down-regulates the inflammatory response and stimulates the host’s production of pro-inflammatory and anti-inflammatory cytokines [20,21]. For example, it was used in the treatment of inflammatory diseases with the VSL#3 probiotic formulation [20,22].

Anti-inflammatory properties of *S. thermophilus* on cell cultures were highlighted. Thus, three strains of *S. thermophilus* (ST1342, ST1275 and ST285) induced anti-inflammatory activity on the promonocytic cell line U937 when co-cultivated with it [23]. The strain ST285 was also shown to induce a significant increase in the expression of anti-inflammatory cytokines IL-4, IL-5 and IL-10, and a decrease in the secretion of pro-inflammatory IL-1β and IFN-γ [24], and was further shown to have immune-modulating effects on peripheral blood mononuclear cells (PBMC) and monocyte cells isolated from PBMC [25,26]. Similarly, Han et al. reported that LPS-treated mouse macrophages co-cultivated with *S. thermophilus* 19 showed a significant decrease in the expression of tumor necrosis factor-α (TNF-α), IL-1β and IL-6 pro-inflammatory cytokines [27].

In our previous study, some strains of *S. thermophilus* such as LMD-9 and CNRZ-21 displayed an anti-inflammatory activity based on high IL-10/IL-12 ratio in the PBMC cell model and reduced IL-8 secretion in the HT-29 cell line [28]. However, the mechanism of action by which this bacterium modulates inflammatory response remains unclear [29]. The overall anti-inflammatory activity of *S. thermophilus* could be due to different actors among which are the surface proteins of bacterial cells. Indeed, these proteins can be substrates for proteases in the gastro-intestinal tract during the digestion process. Peptides with anti-inflammatory activity have already been characterized after hydrolysis of various protein sources of food or endogenous origin [6,30]. Therefore, the anti-inflammatory activity carried by *S. thermophilus* could be mediated, at least in part, by peptides released from these cell surface proteins.

In order to test the validity of this hypothesis, in a first approach, peptides were generated in vitro by tryptic shaving of surface proteins of *S. thermophilus* LMD-9 followed by hydrolysis with trypsin. The hydrolysate was characterized by LC-MS/MS and the anti-inflammatory activity of this one was investigated in cell models using THP-1 human macrophages or intestinal epithelial cells HT-29.

## 2. Materials and Methods

### 2.1. Bacterial Strains and Growth Conditions

*S. thermophilus* LMD-9 (ATCC BAA-491), possessing a cell-wall-anchored protease (PrtS), came from ATCC (American Type Culture Collection, Manassas, VA, USA) collection, and was conserved at −20 °C in sterile reconstituted skim milk (10%, *w*/*v*). The strain was first inoculated at 1% in sterile reconstituted skim milk and incubated overnight at 42 °C to obtain preculture which was then used to inoculate (1%) M17 broth supplemented with 2% of lactose (LM17) [31] and incubated at 42 °C. The growth of strain was followed by measuring the optical density at 650 nm (OD_650nm_).

### 2.2. Enzymatic Shaving of Surface Proteins

Surface proteins of *S. thermophilus* LMD-9 were obtained by shaving approach according to Lecomte et al. 2014 [32]. Briefly, the strain was cultured in LM17 broth with 1% preculture. After 9 h of growth at 42 °C, the culture was used to inoculate at 1% fresh LM17 broth in order to remove all the milk proteins that could be present. Once the culture incubated at 42 °C reached an OD_650nm_ of 4 (end of exponential growth phase), cells were harvested by centrifugation at 3320× *g* for 10 min at 20 °C. The cell pellet was washed thrice with PBS 10 mM (137 mM NaCl, 2.7 mM KCl, 10 mM Na_2_HPO_4_, 2 mM KH_2_PO_4_), pH 7.4. Before shaving their surface proteins with trypsin, the washed cells were concentrated by resuspending them in PBS 10 mM, pH 7.4 to an OD_650nm_ of 30. Afterwards, 10 μg of sequencing grade trypsin (EC 3.4.21.4) from bovine pancreas (Sigma-Aldrich, Saint Quentin Fallavier, France) was added to 1 mL of the concentrated cell suspension and incubated for 1 h at 37°C with shaking (180 rpm). After centrifugation at 10,000× *g* for 10 min at 20 °C and filtration through 0.45 μm filters (Millipore, Molsheim, France), 2 μg of trypsin was added to the supernatant which was incubated for an additional 15 h at 37 °C with shaking (100 rpm). The reaction was stopped by heating the sample at 95 °C for 5 min. The samples were stored at −20 °C until further analysis by LC-MS/MS or lyophilized before evaluation of anti-inflammatory activity. Negative controls containing no trypsin were performed under similar conditions.

### 2.3. LC-MS/MS Analysis

To characterize peptides resulting from surface shaving and subsequent tryptic hydrolysis, mass spectrometry analysis was performed as described previously with some modifications [33]. Preliminary sample concentration step was performed on a Nano-Trap PepMap 100 (C18 column, 75 μm i.d. × 20 mm length, 3 μm particle size, 10 nm pore size; Dionex, Amsterdam, The Netherlands). Separation step was performed as previously described at 35 °C with modifications of the elution gradient. Thus, the gradient started with 5% of solvent B in solvent A to reach 60% of solvent B in 46 min and then 80% of solvent B in 1 min at a flow rate of 0.3 μL/min before re-equilibration of the column. The m/z range used for the recording of mass spectra, the resolution for mass analyzer for MS and MS/MS and the fragmentation step were performed as previously described [33] but time of ion exclusion from fragmentation was set to 20 s. Each sample injection was followed by “blanks” in order to perfectly clean the column and thus avoid “carry-over” problems between two injections. The data was transformed with MSConvert into a mzXML file (peak picking level 1–2) before being interpreted using the software X!TandemPipeline version 0.2.38, e-value < 0.05 per peptide and minimum two peptides per protein. To identify peptides, LMD-9 Proteins fasta file was used. Results were interpreted against Uniprot database available online: https://www.uniprot.org/ (accessed on 15 November 2021) and loctree3 available online: https://rostlab.org/services/loctree3/ (accessed on 15 November 2021).

### 2.4. Anti-Inflammatory Activity of Peptide Hydrolysate on HT-29 Cells

HT-29 cell line (ECACC, Sigma-Aldrich, Saint Quentin Fallavier, France) was maintained in McCoy’s 5A Medium (Gibco, Villebon-sur-Yvette, France) containing 10% heat-inactivated fetal bovine serum (FBS) and 1% streptomycin/penicillin solution at 37 °C in a humidified 5% CO_2_ atmosphere. Confluent cells were washed and trypsinized with 0.25% trypsin-EDTA 1X. Trypsinized cells, prepared in McCoy’s 5A medium at a concentration of 1 × 10^5^ cells/mL, were seeded (500 µL per well) in a 24-well plate and incubated at 37 °C in 5% CO_2_ atmosphere. The medium was changed every 24 h over 4 days. McCoy’s 5A medium used on 4th day and during co-treatment was supplemented with reduced FBS concentration (5%). On the day of the co-incubation, the medium was removed and replaced by 450 µL of McCoy’s 5A medium supplemented with 5% FBS without antibiotics. In each well, 50 µL of either peptide hydrolysate (PH; 0.2–5.0 mg/mL) containing 50 ng/mL of lipopolysaccharide (LPS) from *Escherichia coli* O111:B4 (Sigma-Aldrich, Saint Quentin Fallavier, France) or LPS alone or medium was added to untreated cells. Cells treated with LPS alone were used as reference control. The plate was then incubated at 37 °C in 5% CO_2_ atmosphere for 3 h. Cells were used between passages 18 and 21. Supernatants were collected from the wells and centrifuged at 14,000× *g* for 5 min at 4 °C and the obtained supernatant was conserved at −80 °C until used for IL-8 quantification by ELISA.

### 2.5. Anti-Inflammatory Activity of Peptide Hydrolysate on THP-1 Macrophages

THP-1 cells (ECACC, Sigma-Aldrich, Saint Quentin Fallavier, France) were cultured in Roswell Park Memorial Institute medium (RPMI 1640, Sigma-Aldrich, Saint Quentin Fallavier, France) supplemented with 10% heat-inactivated FBS, 1% sodium pyruvate and 1% penicillin/streptomycin solution in a humidified incubator containing 5% CO_2_ at 37 °C. Culture medium was changed every 2 days. Passage 20 of THP-1 cells was used for anti-inflammatory assay. For anti-inflammatory experiments, THP-1 monocyte cells (8 × 10^5^ cells/mL) in complete culture media containing 20 ng/mL phorbol 12-myristate 13-acetate (PMA; Sigma-Aldrich, Saint Quentin Fallavier, France) were seeded in a 24-well plate (0.5 mL/well). These monocytic cells were differentiated into mature macrophage-like state after 48 h of PMA stimulation. Then, PMA medium was discarded and the differentiated cells were washed twice with pre-warmed PBS. The washed cells were reincubated for another 24 h with 0.5 mL cell culture medium without PMA and antibiotics to let the cells rest after the differentiation step. The differentiated cells were then co-stimulated with 50 ng/mL of LPS and different concentrations of peptide hydrolysate (PH; 0.5, 1.0 and 3.0 mg/mL) for 3 h under similar incubation conditions, whereas cells treated with LPS alone were used as reference control. A positive control was performed using dexamethasone (DEX) solution (10 µM and 25 µM; Sigma-Aldrich, Saint Quentin Fallavier, France) instead of PH during co-treatment with LPS. Finally, the supernatant was collected from the wells, centrifuged at 14,000× *g* for 5 min at 4 °C and stored at −80 °C until used for cytokine quantification by ELISA.

### 2.6. Measurement of Inflammatory Cytokines by ELISA

The concentrations of IL-1β, IL-8 and TNF-α that were released in the collected supernatants from both THP-1 and HT-29 cells were quantified using ELISA kits (Thermo Fisher Scientific, Villebon-sur-Yvette, France) following the manufacturer’s instructions.

### 2.7. Cell Cytotoxicity

The cytotoxicity of PH to THP-1 or HT-29 cells was assessed by measuring lactate dehydrogenase (LDH) activity using an LDH cytotoxicity detection kit (Sigma-Aldrich, Saint Quentin Fallavier, France). The assay measures membrane integrity as a function of the amount of cytoplasmic LDH released into the medium. Briefly, THP-1 or HT-29 cells were prepared as described previously. After 3 h treatment of cells with either LPS, LPS and peptides or LPS and dexamethasone, the supernatants were collected and cytotoxicity was evaluated by measuring LDH activity according to the manufacturer’s instructions. The absorbance was read at 490 and 650 nm, respectively. Control corresponded to supernatants from cells which had not undergone any treatment.

### 2.8. Western-Blotting

After collection of supernatants from THP-1 macrophage culture wells, cells were washed twice with PBS, and then lysed with 100 µL of ice-cold radio-immunoprecipitation assay (RIPA, Thermo Fisher Scientific, Illkirch-Graffenstaden, France) buffer containing Halt Protease and Phosphatase Inhibitor Cocktail (Sigma-Aldrich, Saint Quentin Fallavier, France). Then, protein extracts were spun (14,000× *g*, 4 °C) for 15 min and protein-enriched supernatants were collected and stored at −80 °C until Western blot analysis. The total protein concentration of cell lysates was determined using a BCA protein assay kit (Thermo Fisher Scientific, Illkirch-Graffenstaden, France). After heating at 95 °C for 5 min, protein samples (15 µg/well) were separated by 10% SDS-polyacrylamide gel electrophoresis (PAGE) and transferred to a nitrocellulose membrane (Bio-Rad, Roanne, France). After blocking 1 h at room temperature with Tris-buffered saline Tween 20 (TBST) containing 5% non-fat milk powder, the membranes were treated with primary antibodies (1:1000) for 15 h at 4 °C against IL-1β, COX-2 (RD systems, Rennes, France), and β-actin (Sigma-Aldrich, Saint Quentin Fallavier, France) as a loading control for the quantity and quality of the extracted proteins. After washing with TBST, the membranes were incubated with horseradish peroxidase (HRP)-conjugated secondary antibodies (1:3000) at room temperature for 1 h according to manufacturer’s recommendations (RD systems, Rennes, France). The protein bands were detected by ECL Western Blotting Detection Reagent (GE Healthcare, Buc, France). Results were quantified using Image Lab software (Bio-Rad, Roanne, France). Bands of Pro-IL-1β and COX-2 were normalized to β-actin.

### 2.9. Statistical Analysis

The results of ELISA and Western blot were expressed as mean ± standard error of mean (SEM) from three independent experiments (*n* = 3). Data were subjected to one-way analysis of variance (ANOVA), and the statistical differences between samples were compared using Dunnett’s test. All statistical analyses were carried out with GraphPad Prism 9.0.2 (GraphPad Software, San Diego, CA, USA). A *p*-value of < 0.05 was considered statistically significant.

## 3. Results

### 3.1. Analysis of Surface Proteins of S. thermophilus LMD-9 after Tryptic Shaving

To generate peptides from surface proteins of *S. thermophilus* LMD-9, a shaving approach was applied. This method involves controlled trypsin hydrolysis of the surface proteins of live bacteria followed by a second trypsin hydrolysis of the obtained polypeptides/peptides. Peptides released from surface proteins were identified using mass spectrometry. About 486 proteins were identified by LC-MS/MS analysis. For subsequent analysis, only proteins generating a significant number of peptides (≥14) were considered, resulting in 34 proteins (Table 1). Two proteins, the PrtS protein and the modular protein (2′:3′-cyclic nucleotide 2′-phosphodiesterase) were the most abundant cell surface proteins, a finding supported by the high rate of different peptides obtained from these proteins, i.e., 103 and 156, respectively.

### 3.2. Anti-Inflammatory Effect of Peptide Hydrolysate on HT-29 Cells

**Figure 1:**
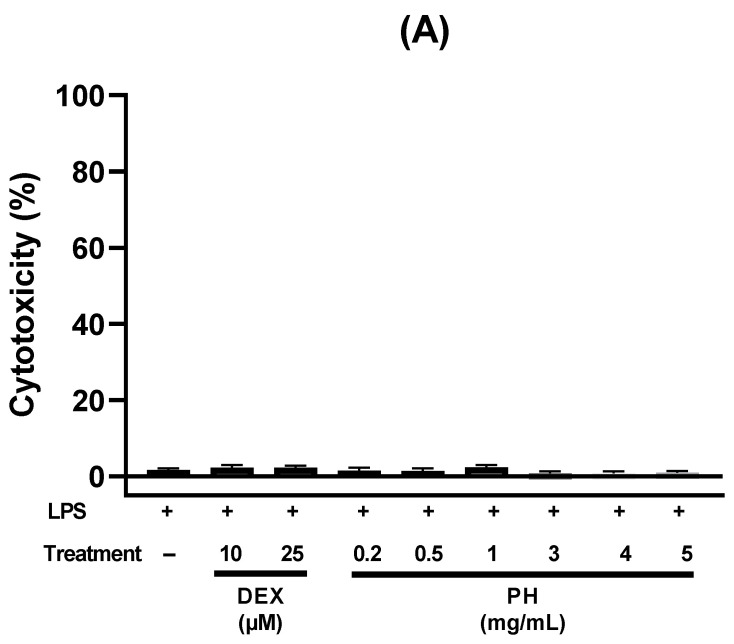

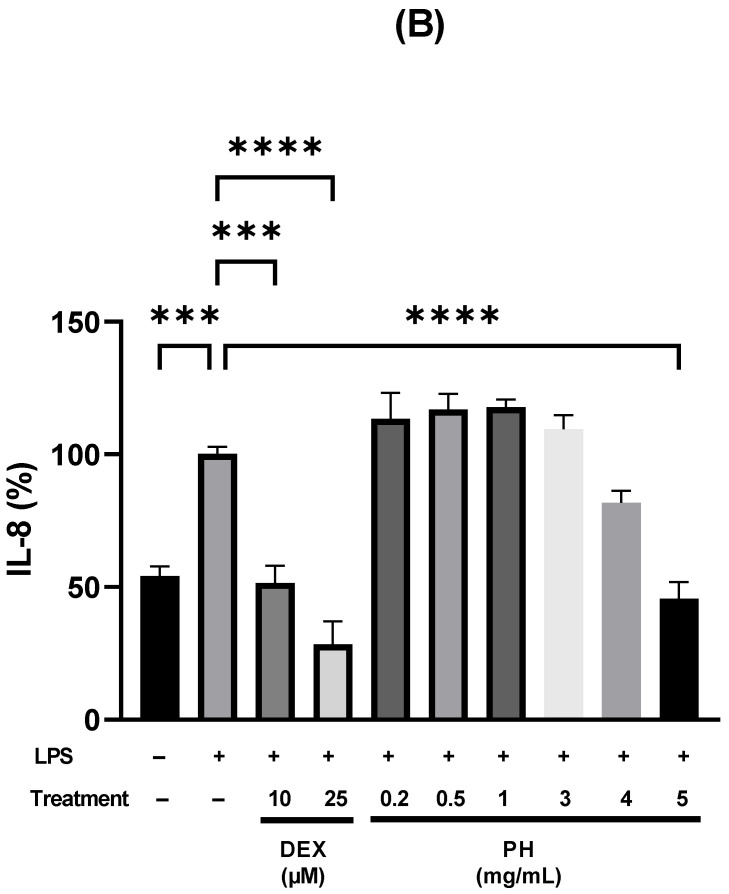
Anti-inflammatory effects of PH at different concentrations (0.2/0.5/1/3/4 and 5 mg/mL) on LPS-stimulated HT-29 cells. (**A**) Cytotoxicity of treatments on HT-29 cells, (**B**) Effect of treatment on IL-8 secretion level. HT-29 cells were incubated in the presence of LPS (50 ng/mL) with and without PH for 3 h. The levels of secreted IL-8 at 3 h in the cell culture medium were analyzed by ELISA. The negative control (LPS−) corresponds to untreated cells. DEX was used as a positive control at 10 and 25 µM. IL-8 (%) is the percentage of IL-8 released by cells compared to its release by those treated with LPS alone. All data are represented as mean ± SEM of 3 independent experiments (*n* = 3). *** *p* < 0.001, **** *p* < 0.0001.

Anti-inflammatory activity of PH was assessed in vitro in the human HT-29 cell line by measuring the concentration of pro-inflammatory cytokine IL-8. For this, HT-29 cells were treated for 3 h with LPS, an inflammatory activator, alone or with LPS and different concentrations of PH (0.2/0.5/1.0/3.0/4.0 and 5.0 mg/mL). LPS-treated cells displayed significantly higher IL-8 secretion levels than untreated cells (*p* < 0.0001; Figure 1B). IL-8 level in LPS-treated cells was used as reference and set to 100%. DEX, a synthetic glucocorticoid, significantly reduced LPS-induced IL-8 production levels by 48.5 and 71.5% at concentration of 10 (*p* < 0.001) and 25 µM (*p* < 0.0001), respectively. Secretion levels of IL-8 in cells treated simultaneously with LPS and PH was dose dependent (Figure 1B). No significant difference in IL-8 levels was observed at concentrations ranging from 0.2 to 4 mg/mL. However, the secretion of IL-8 decreased by 54.5% when PH was used at a concentration of 5 mg/mL. No significant difference in IL-8 decrease was observed between cells treated with 5 mg/mL of PH and those treated with DEX at 10 or 25 µM. LPS, DEX and PH showed no cytotoxic effects since the overall cell viability after 3 h exposure to PH or DEX at all tested concentrations was more than 98% (Figure 1A).

### 3.3. Anti-Inflammatory Effect of Peptide Hydrolysate on THP-1 Macrophages

**Figure 2:**
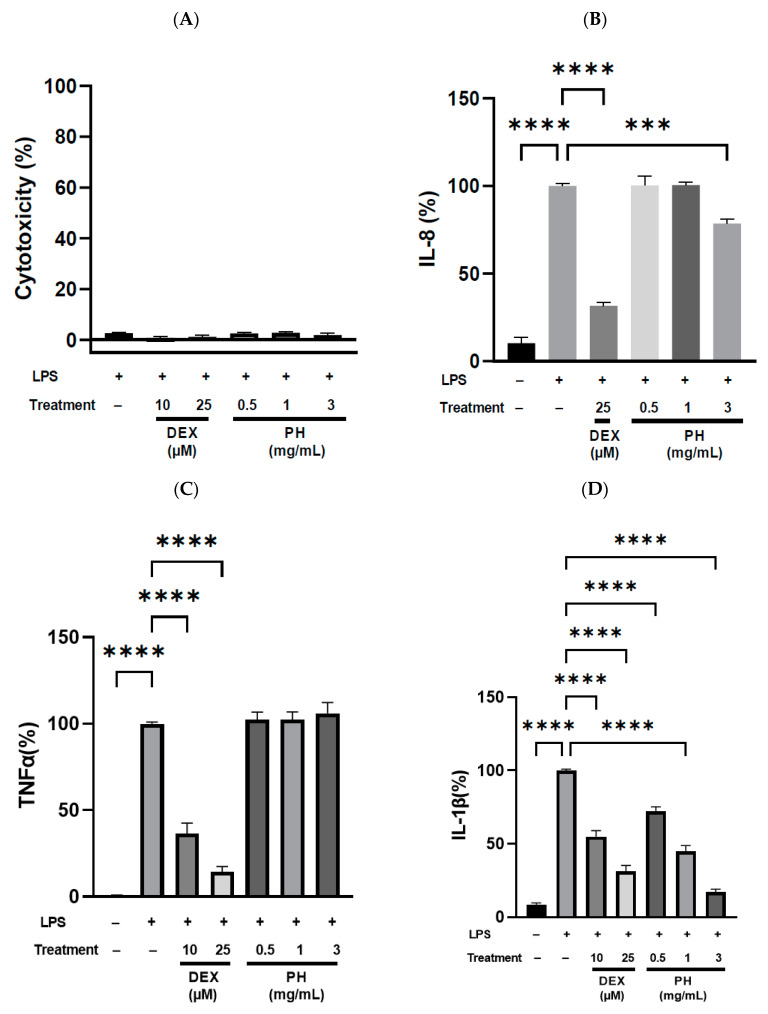
Anti-inflammatory effects of PH at different concentrations (0.5, 1.0 and 3.0 mg/mL) on LPS-stimulated THP- 1 macrophages. (**A**) Cytotoxicity of treatments on THP-1 macrophages, (**B**–**D**) Effect of treatments on IL-8, TNF-α and IL-1β cytokine secretion. THP-1 macrophages were incubated in the presence of LPS (50 ng/mL) with and without PH for 3 h. The levels of secreted TNF-α, IL-1β and IL-8 at 3 h in the cell culture medium were analyzed by ELISA. The negative control (LPS−) corresponds to untreated cells. DEX was used as positive control at 10 and 25 µM. IL-8 (%), TNF-α (%) and IL-1β (%) are the percentages of IL-8, TNF-α or IL-1β, respectively, released by cells compared to their releases by cells treated with LPS alone. All data are represented as mean ± SEM of 3 independent experiments (*n* = 3). *** *p* < 0.001, **** *p* < 0.0001.

The anti-inflammatory activity of PH from *S. thermophilus* was further explored in another in vitro cell model, THP-1 human macrophages. After differentiation, THP-1 macrophages were treated for 3 h with LPS alone or LPS and either DEX or PH (0.5, 1.0 and 3.0 mg/mL). LPS-stimulated cells secreted all three pro-inflammatory cytokines, IL-8, IL1-β and TNF-α, in significantly higher quantities than non-stimulated cells (*p* < 0.0001, Figure 2). Conversely, DEX at 25 µM potently suppressed IL-8, IL-1β and TNF-α release by 68.5, 68.7 and 85.6% (*p* < 0.0001, Figure 2), respectively. A dose-dependent modification in the secretion level of some pro-inflammatory cytokines was observed with PH treatment after stimulation by LPS. The secretion of IL-1β by THP-1 macrophages was significantly reduced following stimulation by LPS and PH treatment. This IL-1β secretion was reduced by 28%, 55% and 83% compared to that of cells treated with LPS alone for PH concentration of 0.5, 1.0 and 3.0 mg/mL, respectively (Figure 2D). Interestingly, no significant difference in the decrease in IL-1β secretion was observed between cells treated with 1 mg/mL of PH and those treated with DEX at 10 µM. Furthermore, PH at 3 mg/mL showed a stronger inhibition of IL-1β secretion (83%) than DEX at both 10 and 25 µM (45.2% and 68.7%, respectively; Figure 2D). Treatment of LPS-stimulated THP-1 macrophages by PH at 0.5 or 1.0 mg/mL did not result in a statistically significant reduction in IL-8 secretion relative to cells treated with LPS alone (Figure 2B). However, PH added at 3.0 mg/mL inhibited IL-8 secretion by 21.5%. Secretion of the third cytokine evaluated, TNF-α, was unaffected by PH treatment at the concentrations previously tested for secretion of IL-8 and IL-1β (Figure 2C). As observed with HT-29 cells, no significant cytotoxic effects were detected regardless of the treatments used since viability of THP-1 macrophages was higher than 98% (Figure 2A).

**Figure 3:**
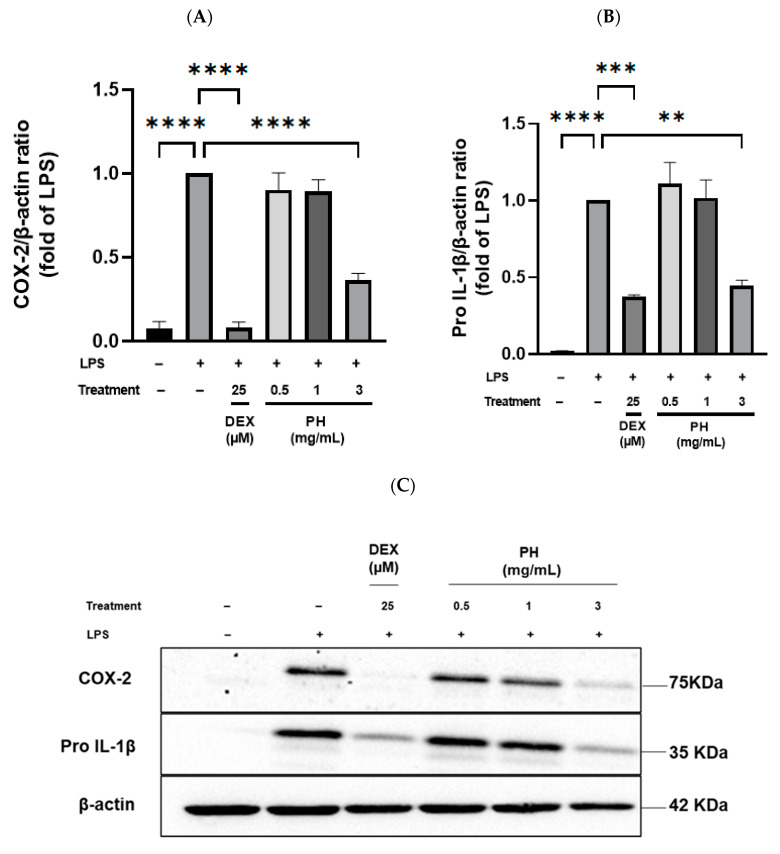
Effects of PH on COX-2 and Pro-IL-1β protein expression in LPS-stimulated THP-1 macrophages. (**A**–**C**) THP-1 macrophages were incubated in the presence of LPS (50 ng/mL) with or without PH at 0.5, 1.0 or 3.0 mg/mL for 3 h. DEX was used as a positive control at 25 µM. Protein content (15 µg) of each sample was loaded on 10% SDS-PAGE. The results were calculated as a relative intensity to the β-actin. All data are represented as mean ± SEM of 3 independent experiments (*n* = 3). **p < 0.01, *** *p* < 0.001, **** *p* < 0.0001.

To explore the potential mechanism by which PH attenuated inflammation in THP-1 macrophages, expression of inflammation-related proteins, especially cyclooxygenase-2 (COX-2) and Pro-IL-1β, was assessed by Western blot. Treatment of cells with LPS alone for 3 h increased significantly (*p* < 0.05) the production of pro-inflammatory COX-2 and Pro-IL-1β proteins (Figure 3). DEX used at 25 µM in LPS-stimulated THP-1 macrophages significantly reduced expression of COX-2, bringing it back to a basal expression, and of Pro-IL-1β. Treatment of LPS-stimulated cells with 0.5 or 1 mg/mL of PH did not affect the expression of both proteins. On the contrary, treatment with 3 mg/mL of PH significantly decreased the expression of these pro-inflammatory mediators (Figure 3A,B). The reduction in expression with 3 mg/mL of PH was of 63.6% and 55.3% for COX-2 and Pro-IL-1β, respectively. Of interest, no significant difference was observed in the expression level of Pro-IL-1β between LPS-stimulated cells treated with 25 µM DEX or 3 mg/mL PH.

## 4. Discussion

LAB are among the most intensively exploited microorganisms in the dairy industry. They are ingested with the food product they have made possible, and their regular consumption has now been reported to affect consumer health positively. An increased attention is given to their anti-inflammatory effects. Thereby, LAB isolated from kimchi showed an anti-inflammatory effect on acid-induced acute colitis in model mice [12]. Gao et al. (2019) showed that the heat-killed *S. thermophilus* decreased the inflammatory markers such as LPS, IL-6 and TNF-α and increased the anti-inflammatory interleukin IL-10 in Zucker diabetic fatty rats [34]. Junjua et al. 2016 reported in vitro an anti-inflammatory activity of various live *S. thermophilus* strains [28]. Thus, *S. thermophilus*, like other LAB, is able to display anti-inflammatory activity in both live and heat-inactivated forms [28,35].

Major cell wall components of Gram-positive bacteria such as peptidoglycan (PGN) or lipoteichoic acid (LTA) show immunomodulatory properties [36]. *Lactiplantibacillus plantarum* LTA down-regulates IL-8 production in human intestinal epithelial Caco-2 cells [37]. Fernandez et al. 2011 showed that PGN derived from lactobacilli decreased inflammatory responses in the colon [38]. These studies suggest that a link exists between cell components of lactobacilli and their anti-inflammatory potential. The mechanisms by which *S. thermophilus* exerts anti-inflammatory activity are not well understood and it is possible that surface bacterial components may be involved. This bacterium is ingested and the proteins that are present on its surface can undergo proteolysis during digestion. The constitutive proteins of foods and endogenous proteins of the digestive tract undergo proteolysis processes during digestion to allow the absorption of nutrients of protein origin. Many studies have shown that peptides potentially released during digestion have biological activities that could modulate consumer physiology, some of which display anti-inflammatory properties. Therefore, peptides derived from the digestive proteolysis of extracellular proteins of *S. thermophilus* may contribute to the overall anti-inflammatory effects of this bacterium as they could be the first bacterial components to interact with the host. Thus, in the present study, in vitro anti-inflammatory activity of a peptide hydrolysate obtained from surface proteins of *S. thermophilus* LMD-9 was investigated. In a first approach, the peptide hydrolysate (PH) was obtained by shaving the surface of living bacterial cells with trypsin followed by a trypsinolysis. Trypsin is a pancreatic endoprotease with a narrow specificity, which specifically cleaves the peptide bonds at the C-terminal side of arginine or lysine residues [39] and generates peptides with an average size of 700–1500 Da, which are in the ideal range for MS analysis. Furthermore, the presence of positively charged residues in peptide sequences facilitates ionization and consequently detection of peptides by MS analysis [40]. Trypsin can diffuse into the cell wall and interact with the membrane and cell-wall-integrated proteins. Surface proteins can be mainly constituted of integral membrane proteins, lipoproteins covalently attached to membrane lipids, proteins with the LPXTG motif covalently attached to peptidoglycan, non-covalently bound proteins, and moonlighting proteins [41,42]. Thus, the majority of peptides that were identified after shaving belongs to the surface proteins of *S. thermophilus* LMD-9, yet a few corresponding to cytoplasmic proteins have also been identified (Table 1). The presence of such peptides is not unexpected and could be explained either by a slight cell lysis during shaving or by the presence of intracellular/surface moonlighting proteins such as metabolic enzymes, e.g., enolase [32], and intracellular chaperons (GroEL/DnaJ), as demonstrated by Mu et al. (2020) that the enolase EnoM from *S. thermophilus* could bind to the cell surface [43]. On the other hand, intracellular chaperons could be found in surface proteomes because they have a transient interaction with the membrane or with proteins associated with the membrane to perform secondary functions [44].

The present study demonstrated that the surface proteins of *S. thermophilus* could be a source of anti-inflammatory peptides when hydrolyzed by trypsin since an anti-inflammatory activity of the hydrolysate obtained after shaving of *S. thermophilus* LMD-9 was highlighted in two different cell models, i.e., HT-29 and THP-1 macrophages. HT-29, a human colorectal adenocarcinoma cell line which mimics characteristics of mature intestinal epithelial cells, is commonly used in studies related to inflammatory bowel disease [45]. On the other hand, the THP-1 cell line has been widely used to study immune responses since cells can be used in the monocyte state as well as in the macrophage-like state after differentiation. THP-1 macrophages are a unique, sensitive and precise model used to explore the anti-inflammatory activity of various drugs as well as bioactive peptides [46,47]. Inflammatory response of these cell types can occur when THP-1 macrophages or HT-29 cells are stimulated with LPS [48,49]. 

Anti-inflammatory activity of PH was evaluated by quantifying pro-inflammatory cytokines (TNF-α, IL-1β and IL-8) because they play a pivotal pathological role in several inflammatory diseases [50]. First of all, it must be noted that PH showed no cytotoxic effect for all the concentrations tested regardless of the cell model used. The results obtained with the HT-29 cell model showed that PH treatment with at least 4 mg/mL significantly reduced the secretion of IL-8 in LPS-stimulated cells. IL-8 cytokine is an important pro-inflammatory mediator secreted by both intestinal cells and activated macrophages [51]. It is also one of the predominant cytokines secreted from epithelial cells and its production is important in HT-29 cells [52,53]. Previous studies have reported that surface-layer protein B (SlpB) mediated the adhesion of *Propionibacterium freudenreichii* CIRMBIA129 to intestinal epithelial HT-29 cells [54] and reduced IL-8 expression in LPS-induced HT-29 cells [55]. Li et al. (2011) showed that surface-layer protein (Slps) of *Lactobacillus acidophilus* decreased IL-8 secretion in Caco-2 cells stimulated by *Salmonella Typhimurium* [56].

Results showed that the treatment of THP-1 macrophages with LPS induced an increased secretion of the pro-inflammatory cytokines TNF-α, IL-1β and IL-8 as well as COX-2 and Pro-IL-1β expression. The co-treatment of LPS-stimulated THP-1 macrophages with PH at a concentration of 3 mg/mL displayed an anti-inflammatory activity since it significantly decreased the production of the IL-1β and IL-8 cytokines, and the expression of COX-2 and Pro-IL-1β. The reduced expression of pro-inflammatory enzyme COX-2 observed in PH-treated macrophages in presence of LPS can be explained by the reduction in IL-1β secreted by macrophage. A direct relation between secretion/expression of IL-1β and COX-2 has been proposed in many studies. For example, it is reported that IL-1β induced the expression of COX-2 in breast cancer cells [57]. Despite the decrease in many inflammatory parameters in LPS-stimulated THP-1 macrophages after co-treatment with PH, TNF-α secretion remained unchanged. This result was consistent with a previous study showing that phenolic-rich extracts from common beans downregulated IL-1β and IL-6 secretion but were unable to suppress TNF-α mRNA expression in LPS-stimulated RAW 246.7 macrophages [58]. On the other hand, dexamethasone in our conditions significantly decreased TNF-α secretion in LPS-stimulated macrophages THP-1.

Regardless of the cell model, our results showed an anti-inflammatory activity of a PH obtained after tryptic shaving of surface proteins of *S. thermophilus* LMD-9 that has never been observed previously to the best of our knowledge. However, could the level of active concentration of PH found be consistent with an action in the context of a normal diet? One mL of a 125 g yogurt generally contains about 8.10^8^ viable *S. thermophilus* cells [59]. By making an approximation using the *E. coli* model, a Gram-negative bacterium which weighs about 0.727 pg and that contains 70% water and 55% protein on a dry matter basis [60], the consumption of one yogurt might provide about 12 mg of protein to the consumer. By referring to this amount, it can be assumed that the active concentrations of PH, i.e., 0.5 to 3 mg/mL are at a compatible level. Thus, peptides released by digestive proteolysis from surface proteins could participate in part in the anti-inflammatory effect of *S. thermophilus*. The anti-inflammatory effect could come from one peptide fragment of the hydrolysate or result from a synergistic effect of several peptide fragments. Several dipeptides, i.e., YG from bovine κ-casein [61]; FL, LL and MK from ovotransferrin [62]; AL, IA and VH from velvet antler protein [63]; and LF from bovine β-lactoglobulin [64] have been shown to exert anti-inflammatory activities. These dipeptides are present in some proteins belonging to the proteome of *S. thermophilus*. According to Proteome pI database [65], these eight dipeptides represent 3.7% of the 400 possible theoretical combinations that can be released from the proteome of *S. thermophilus*, a percentage higher than that which can be calculated assuming that each dipeptide exists with the same probability (2%). These eight dipeptides were found in numerous peptide sequences generated after tryptic shaving of the LMD-9 strain. Moreover, it has already been shown that a mixture of peptides (PepMix) exhibited greater anti-inflammatory activity than any individual peptide at the same concentration [63]. The highlighted anti-inflammatory activity could come from peptide(s) generated from the PrtS protein and/or from the modular protein (2′:3′-cyclic nucleotide 2′-phosphodiesterase), considering the large number of different peptides obtained from these proteins, the high coverage rate of these sequences and the abundance of anti-inflammatory dipeptides cited above in these sequences.

## 5. Conclusions

To our knowledge, this is the first study that provides experimental evidence of an anti-inflammatory potential of a hydrolysate derived from tryptic shaving of surface proteins of *S. thermophilus* LMD-9. The peptides obtained by trypsin hydrolysis after shaving exhibited anti-inflammatory activities in HT-29 cells as well as THP-1 macrophages stimulated by LPS, by modulation of pro-inflammatory mediators including IL-8 and IL-8, IL-1β and COX-2, respectively. Hence, the modulation of inflammatory response by this bacterium could be due to the release of surface peptides during digestion, and this microorganism could be used as a functional food ingredient or as a preventive agent against low-grade inflammation.

However, further research should be undertaken to verify if a more complex digestive system is still able to release the active fragment(s) and if the release is strain dependent.

## Figures and Tables

**Table 1:** *S. thermophilus* LMD-9 surface proteins identified by LC-MS/MS after trypsin shaving followed by tryptic hydrolysis.

Protein ID	New Locus Number	Description	MW (kDa)	Location	Coverage (%)	Nb Specific Sequences Identified
Nucleotide metabolism and transport						
STER_1992|ID:1900614|guaB|	STER_RS09740	IMP dehydrogenase	52.88	Cyto	56.7	17
STER_1845|ID:1900521|	STER_RS09015	DNA-directed RNA polymerase subunit beta	133.17	Cyto	32.3	29
STER_0198|ID:1898877|	STER_RS00970	2′:3′-cyclic-nucleotide 2′-phosphodiesterase (modular protein)	91.21	CS	64.9	156
Post-translational modification, protein turnover, chaperone function						
STER_0846|ID:1899371|	STER_RS04165	exported protein of unknown function (subtilisin-like serine protease PrtS)	173.05	CS	51.3	103
STER_1578|ID:1900309|clpB|	STER_RS07755	protein disaggregation chaperone	77.15	Cyto	69.0	70
STER_0253|ID:1898921|groL|	STER_RS01230	Cpn60 chaperonin GroEL, large subunit of GroESL	56.89	Cyto	69.8	32
STER_0163|ID:1898844|dnaK|	STER_RS00790	chaperone Hsp70, co-chaperone with DnaJ	64.76	Cyto	50.8	27
STER_0191|ID:1898870|tig|	STER_RS00935	peptidyl-prolyl cis/trans isomerase (trigger factor)	46.65	Cyto	61.9	26
STER_2002|ID:1899687|degP|	STER_RS09790	serine endoprotease (protease Do), membrane-associated	42.77	CM/M	72.3	37
STER_0014|ID:1898716|ftsH|	STER_RS00070	protease, ATP-dependent zinc-metallo	71.95	M	34.8	16
STER_0648|ID:1899776|clpA|	STER_RS03195	ATPase and specificity subunit of ClpA-ClpP ATP-dependent serine protease, chaperone activity	83.69	CM	24.4	13
Translation						
STER_0524|ID:1899138|tufB|	STER_RS02570	protein chain elongation factor EF-Tu (duplicate of tufA)	43.84	Cyto	68.9	35
STER_1762|ID:1900456|fusA|	STER_RS08620	protein chain elongation factor EF-G, GTP-binding	76.56	Cyto	59.4	33
STER_0639|ID:1899231|	STER_RS03135	40S ribosomal protein S1	43.88	Cyto	50.4	22
STER_1844|ID:1900520|rpoC|	STER_RS09010	RNA polymerase, beta prime subunit	135.16	Cyto	21.4	21
STER_1526|ID:1900271|deaD|	STER_RS07510	ATP-dependent RNA helicase	58.96	CM	44.5	21
STER_1904|ID:1900567|rplB|	STER_RS09330	50S ribosomal subunit protein L2	29.91	Cyto	49.6	19
STER_0383|ID:1899016|infB|	STER_RS01860	conserved protein of unknown function (translation inibition factor IF-2)	103.73	Cyto	20.3	17
STER_0105|ID:1898797|rpsB|	STER_RS00525	30S ribosomal subunit protein S2	28.40	Cyto	69.1	17
STER_0247|ID:1898915|proS|	STER_RS01210	prolyl-tRNA synthetase	68.48	Cyto	35.2	16
STER_1893|ID:1900556|rpsH|	STER_RS09275	30S ribosomal subunit protein S8	14.78	Cyto	58.7	15
Carbohydrate metabolism and transport						
STER_1761|ID:1900455|gapA|	STER_RS08615	glyceraldehyde-3-phosphate dehydrogenase A	36.00	Cyto	83.4	28
STER_0684|ID:1899266|eno|	STER_RS03365	enolase	46.95	CS/M/Cyto	63.2	28
STER_1163|ID:1899979|pykF|	STER_RS05740	pyruvate kinase I	54.49	Cyto	55.1	22
STER_1172|ID:1899988|gpmA|	STER_RS05785	phosphoglyceromutase 1	26.17	Cyto	53.7	21
STER_1876|ID:1900541|kbaY|	STER_RS09185	tagatose 6-phosphate aldolase 1, kbaY subunit	31.51	Cyto	47.3	14
STER_0241|ID:1898909|	STER_RS01180	glucose-6-phosphate isomerase A (GPI A)	49.76	Cyto	34.7	14
STER_1241|ID:1900050|gabD|	STER_RS06125	succinate-semialdehyde dehydrogenase I, NADP-dependent	50.79	Cyto	45.0	18
STER_1755|ID:1900453|pgk|	STER_RS08580	phosphoglycerate kinase	42.21	Cyto	58.8	20
STER_0895|ID:1899407|	STER_RS04435	putative ribulose-phosphate 3-epimerase	58.13	Cyto	34.8	15
Cell wall/membrane/envelop biogenesis						
STER_0042|ID:1898743|	STER_RS00210	secreted 45 kDa protein precursor	46.45	CS	45.6	17
Amino acid transport and metabolism						
STER_1411|ID:1900180|	STER_RS06940	putative transporter subunit: periplasmic-binding component of ABC superfamily	72.18	CS	47.3	22
Unknown function						
STER_0576|ID:1899177|	STER_RS02840	mucus-binding protein precursor (fragment)	108.40	CS	29.1	31
STER_0856|ID:1899377|	STER_RS04220	CD4+ T-cell-stimulating antigen precursor	37.62	CS	53.2	14

CS: cell surface protein; M: membrane located protein; CM: cell membrane located protein; Cyto: cytoplasmic protein.

## Data Availability

Data is contained within the article.
